# First Insights into the Occurrence of Circular Single-Stranded DNA Genomes in Asian and African Cattle

**DOI:** 10.3390/ani13091492

**Published:** 2023-04-27

**Authors:** Marie-Thérèse König, Kai Frölich, Anabell Jandowsky, Tobias Knauf-Witzens, Christoph Langner, Richard Dietrich, Erwin Märtlbauer, Andrea Didier

**Affiliations:** 1 Department of Veterinary Sciences, Institute of Food Science, Faculty of Veterinary Medicine, Ludwig-Maximilians-Universität München, Schönleutnerstraße 8, 85764 Oberschleißheim, Germany; 2 Tierpark Arche Warder, Zentrum für Seltene Nutztierrassen e. V., Langwedeler Weg 11, 24646 Warder, Germany; 3 Wilhelma Zoological-Botanical Gardens Stuttgart, Wilhelma 13, 70376 Stuttgart, Germany; 4 Stralsund Zoological Garden, Grünhufer Bogen 2, 18437 Stralsund, Germany

**Keywords:** cattle, BMMF, *Cressdnaviricota*, *Genomoviridae*, circular ssDNA, human cancer, indirect carcinogenesis

## Abstract

**Simple Summary:**

Since 2014, a special class of meat and milk-derived circular DNA agents—termed bovine meat and milk factors (BMMF)—has been linked to the process of indirect carcinogenesis in humans. These BMMF include members of the virus family *Genomoviridae*, as well as unclassified DNA elements exhibiting both viral and plasmid attributes. Initial detection of BMMF in European cattle samples led to the assumption that BMMF are exclusively present in aurochs-derived cattle and consequently in food produced thereof. In the meantime, a more widespread occurrence including water buffalo, sheep and goat milk has been shown. The aim of the present study was to broaden our knowledge concerning the BMMF occurrence in different and less domesticated ruminants like yaks, zebus and watusi cattle that serve for food production in various countries. The predominant detection of BMMF in fecal matter compared to blood provides novel clues regarding the origin and transmission routes of these entities finally reaching the food chain.

**Abstract:**

Circular replicase-encoding single-stranded (CRESS) DNA viruses and other circular DNA agents are increasingly found in various samples and animals. A specific class of these agents—termed bovine meat and milk factors (BMMF)—has been supposed to act as a factor in indirect carcinogenesis in humans. Initial observations attributed the BMMF to European cattle breeds and foodstuffs produced thereof. In the present study, blood and fecal samples from African and Asian cattle were examined. BMMF molecules and genomoviruses were detected in all bovids under study. The majority (79%) of the 29 circular elements could be assigned to BMMF groups 1 and 2, whereas CRESS viruses of the family *Genomoviridae* accounted for the smaller part (21%). Two genomoviruses belong to the genus *Gemykibivirus* and one to the genus *Gemykrogvirus*. The remaining three might be considered as novel species within the genus *Gemycircularvirus*. The majority of all isolated molecules originated from fecal samples, whereas only three derived from blood. The results from this study expand our knowledge on the diversity and presence of circular DNA in different ruminants that serve for food production in many countries over the world.

## 1. Introduction

Beside European cattle, food originating from Asian and African cattle play an important role in human nutrition regarding worldwide milk and meat consumption [1,2,3,4]. Therefore, monitoring animal health status is a prerequisite not only to prevent the spread of zoonoses, but also to identify new, potentially pathogenic agents at an early stage.

Novel circular DNA entities—known as BMMF (bovine meat and milk factors)—have been isolated from multiple bovine samples [5]. Initially, four different BMMF groups were distinguished by their DNA homologies and functional elements [5,6]. Members of BMMF groups 1 and 2 resemble the Sphinx 1.76 and Sphinx 2.36 DNAs that were originally co-purified from transmissible spongiform encephalopathy (TSE) particles [7]. They further exhibit homologies to an *Acinetobacter baumannii* plasmid and an *Acinetobacter* sp. phage DNA. Molecules assigned to BMMF group 3 were classified as novel Gemycircularviruses [5,8], members of the virus family *Genomoviridae* [9]. BMMF group 4 consists of a single isolate sharing highest sequence similarities to a *Psychrobacter* sp. plasmid [10].

In silico analyses revealed that BMMF are heterogeneous, circular single-stranded (ss) DNA elements encoding at least a putative replication protein called Rep [5,11,12]. Rep enables DNA amplification by rolling circle replication (RCR). This replication mechanism was originally described in *Escherichia coli* bacteriophage ΦX174 [13,14]. Since then, the group of DNA entities harnessing RCR is constantly growing, now comprising bacteriophages, plasmids of Gram-positive and Gram-negative bacteria, archaeal plasmids and eukaryotic viruses [15,16,17]. One important virus family utilizing this replication mechanism is the family *Genomoviridae* that belongs to the newly established phylum *Cressdnaviricota* [18,19]. Circular replicase-encoding single-stranded (CRESS) DNA viruses have been isolated from a multitude of environmental-and animal-associated sources, often comprising fecal matter [20,21,22,23,24,25,26]. The high similarity of genomovirus replication proteins to Reps of plant-infecting geminiviruses points to a close evolutionary relationship [27]. From a functional point of view, mainly two types of Reps are distinguishable: firstly, Reps exhibiting endonuclease activity only and thus necessitating an additional host-encoded helicase [28]; and secondly, Reps encompassing an endonuclease as well as a helicase domain. These bifunctional Reps are characterized by conserved amino acid (aa) motifs which are the N-terminal motifs I, II and III—typical for endonucleases—and the C-terminal Walker A, Walker B and Walker C motifs, necessary for the helicase function. Genomoviruses have further typical virus-like properties due to the presence of a capsid protein-coding region [23]. These viruses differ markedly from BMMF1, 2 and 4, which remain unclassified. BMMF constitutes a novel class of agents sharing characteristics of both known viruses, as well as bacterial plasmids [5].

Since the first isolations of BMMF from cattle blood, serum, milk and additionally brain tissue from multiple sclerosis-affected humans in 2014 [8,10,11,29], a discussion has arisen about whether these entities are solely present in *Bovidae* of taurine origin. An answer to this question is of utmost importance, as BMMF and BMMF translation products—especially Rep—have been detected in different human cancer types (e.g., colon, breast, lung and pancreatic cancer). These studies further showed the induction of an inflammatory response potentially contributing to indirect carcinogenesis [30,31,32,33,34]. Recent investigations revealed that BMMF molecules are not exclusively present in aurochs-derived taurine cattle but are also detectable in the milk and blood of water buffaloes, sheep and goats [35,36,37]. From a phylogenetic point of view, these species are only distantly related to taurine cattle. In addition to bovine specimens, fragments of BMMF-like DNA have also been detected in various animal and non-animal derived foods [38].

However, further bovids like yak, zebu and watusi cattle, which are also part of worldwide livestock, have not been assayed so far. Multitudinous flocks of these cattle are kept in Asia, South America and Africa to serve as a source of food for humans. Therefore these cattle might represent a potential source regarding the ingestion of BMMF1, 2 and 4 and additionally CRESS DNA viruses [39,40]. Thus, the current study was initiated to expand our knowledge concerning the distribution of BMMF and genomoviruses and to obtain new insights into the variety of sources from which these molecules might enter the food chain.

## 2. Methods

### 2.1. Sample Collection

The sample-set comprised sera, EDTA-stabilized blood and fecal matter from yaks, zebus, watusi cattle and water buffaloes housed in different German zoos (Table 1). Blood and serum samples were collected during the mandatory, routine blood sampling for bovine herpes virus-1 diagnostics. Thus, an additional permit to conduct this study was not required. After collection, all samples were shipped cooled except for serum, which was shipped frozen.

### 2.2. DNA Extraction and RCA

The QIAamp Mini Kit (Qiagen, Hilden, Germany) served for DNA isolation from serum and EDTA-stabilized blood. Sample volume was 200 µL, and DNA extraction was carried out according to the user manual. The QIAamp PowerFecal DNA Kit (Qiagen, Hilden, Germany) was applied for DNA extraction from 250 mg of fecal matter according to the manufacturer’s instructions. A DeNovix DS-11 FX (DeNovix, Wilmington, DE, USA) spectrophotometer served for determination of DNA yield and quality (i.e., OD260/OD280 ratio). After DNA extraction, approx. 40 ng DNA of each sample was applied for rolling circle amplification (RCA) by using the TempliPhi Amplification Kit (Cytiva, Marlborough, MA, USA) with random primers, according to the manufacturer’s instructions. For optimal amplification of circular DNA, samples were incubated at 30 °C for 18 h.

### 2.3. PCR Screening and Recovery of Full-Length Sequences

After completion of RCA, all samples underwent a PCR procedure for the detection of short-size BMMF amplicons (approx. 300–600 bp). The applied primers, PCR reaction set-ups and thermal programs have been published elsewhere [35,36]. In case of positive results, primer pairs for inverse PCR were designed based on the sequences of short fragments, followed by amplification of full-length sequences. Full-length amplicons were cloned into pCR2.1 TOPO TA vector (ThermoFisher Scientific Inc., Waltham, MA, USA). After transformation of *E. coli*, MiniPrep and restriction digest, insert-bearing clones were subjected to Sanger Sequencing on both strands. MWG Eurofins (Ebersberg, Germany) operated all sequencing reactions.

### 2.4. Data Analysis

For the characterization and annotation of all full-length sequences, several bioinformatics tools were applied. Firstly, sequences were subjected to BlastSearch against the nucleotide database at NCBI [41]. The MEGA software (v. 10.2.6, Kumar, Stecher, Li, Knyaz, and Tamura, The Pennsylvania State University, PA, USA) was applied to generate multiple sequence alignments and phylogenetic trees by the means of the Maximum likelihood method. Bootstrap values were computed with 500 replicates [42]. The DNASTAR MegAlign Pro software version 17.1.1 (DNASTAR, Inc., Madison, WI, USA) was used for computing multiple sequence alignments with MUSCLE and pairwise homologies of all BMMF elements [43,44]. To display the pairwise genome identity scores of novel genomoviruses, the Sequence Demarcation Tool Version 1.2 (SDTv1.2) was utilized [45]. Tandem repeats (TR) with a maximum size of 30 nucleotides (nt) [46] and inverted repeats (IR) [47] upstream of *rep*-gene sequences were checked using Emboss explorer. The minimum size of IR was set to 4 nt. ORFfinder software (version 1.3.0, National Library of Medicine, Bethesda, MD, USA) at NCBI served for in silico detection of potential open reading frames (ORFs) allowing ‘ATG’ initiation only [48]. If no ORFs were found based on ‘ATG’ as the start codon, alternatives were permitted. The further comparative and functional analysis included only ORFs ≥ 60 aa. Illustrations of circular DNA elements were plotted with SeqBuilder Pro DNASTAR software (Lasergene Inc., v. 17.1. DNASTAR. Madison, WI, USA) [43]. Annotated genomes from this study were deposited in the GenBank database (Accession numbers OQ633422-OQ633450).

## 3. Results and Discussion

**Figure 1:**
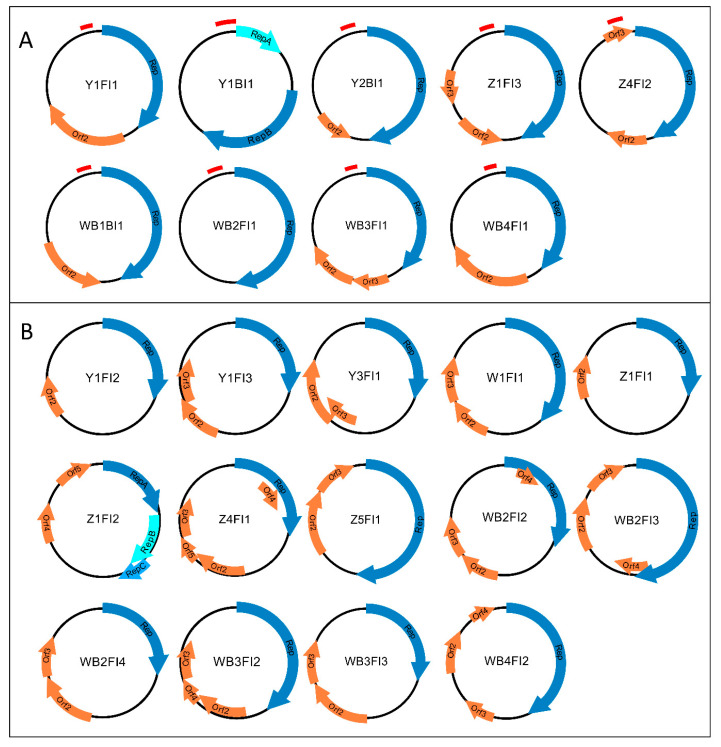
Schematic illustration of 23 BMMF molecules identified in this study. The arrows in different shades of blue represent potential *rep*-genes. Orange arrows indicate uncharacterized ORFs. All elements belonging to the BMMF group 1 (**A**) additionally feature a tandem repeat region upstream of the *rep*-gene that is illustrated by a red bar. Molecules belonging to the BMMF group 2 (**B**) lack this specific attribute.

The present study aimed to detect circular ssDNA elements in different bovids like yak, zebu, water buffalo and watusi cattle and to assort them to either the BMMF groups or the CRESS viruses. In reviewing the literature, no data were found on the occurrence of BMMF or CRESS viruses in these animals descending from African and Asian progenitors, except for one study in which genomoviruses were isolated from water buffaloes kept in Switzerland [37]. The aforementioned animals in human care contribute to the food supply of the population in many countries on different continents. Representatives of these species living in Europe are mainly housed in zoos and wildlife parks. These zoo animals do not come into close daily contact with their caretakers, e.g., during routine milking, as with livestock. In our study, fecal matter and blood samples (routinely collected for BHV-1 serology) served for DNA isolation, RCA and BMMF amplification. This procedure resulted in the detection of 29 circular ssDNA molecules (Figure 1), most of which showed highest similarities to BMMF1 and 2, as first described by zur Hausen et al. and de Villiers et al. [5,6,11,12,29]. The smaller fraction of the isolated elements belonged to the *Genomoviridae* virus family. Except for three circular DNAs detected in EDTA-blood, the majority (26) of all molecules was isolated from feces. An overview of all entities discovered in blood and feces is given in Table 2. The corresponding Blast Search hits are listed in Supplementary Table S1.

### 3.1. Fecal Samples

#### 3.1.1. Isolation of BMMF Molecules from Feces

To assess the extent to which feces may contribute to the distribution of BMMF, 16 individual fecal samples from yaks, zebus, water buffaloes and watusi cattle from three different German zoos were assayed. We demonstrated that BMMF were present in fecal samples of all four species under study. Overall, 20 BMMF molecules were isolated from nine individual fecal samples. Interestingly, individual fecal samples included up to four different circular DNA molecules. This clearly contrasts the results obtained from bovid milk samples in which a maximum of two sequences could be isolated per individual using the same methodological approach [35].

**Figure 2:**
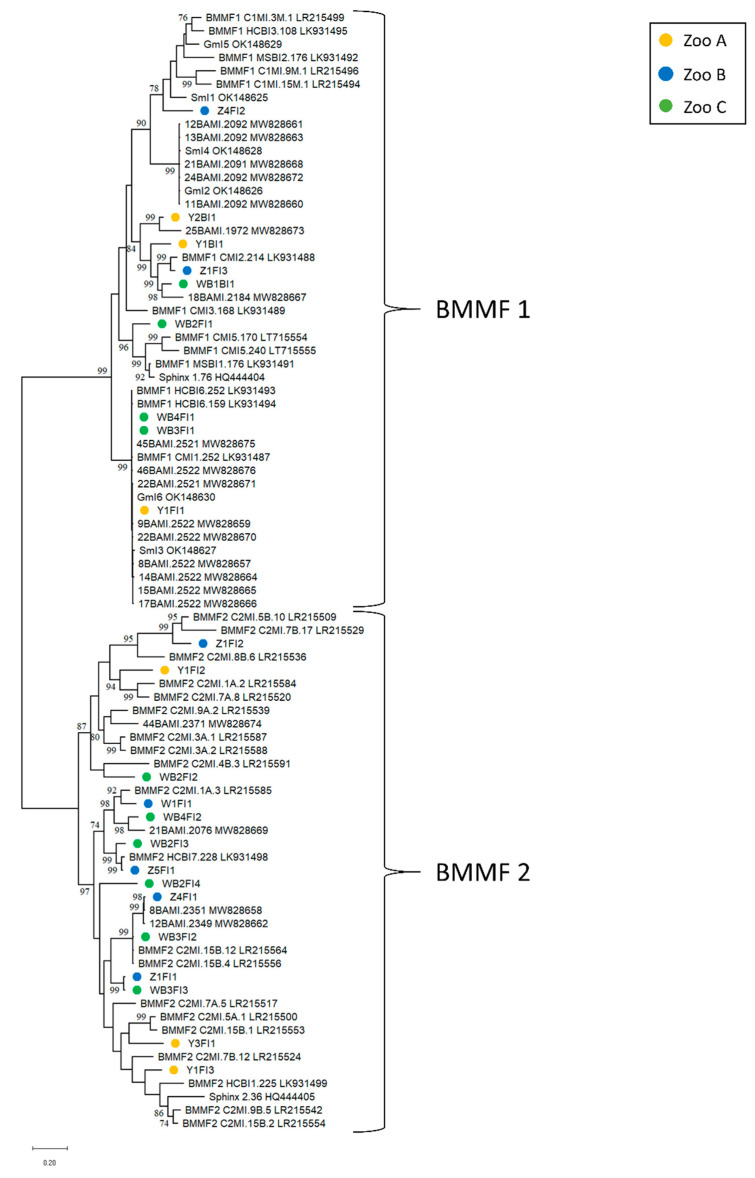
Maximum Likelihood phylogenetic tree of BMMF molecules detected in this study, as well as representative BMMF1 and 2 isolates, Sphinx 1.76, Sphinx 2.36 and related BMMF molecules isolated from water buffalo, sheep and goat milk. The evolutionary history was inferred by using the Maximum Likelihood method and the Tamura-Nei model [49]. The bootstrap consensus tree is based on 500 replicates. The percentage of trees in which the associated taxa clustered together is shown next to the branches. Branch support values lower than 70% were not included. Colored dots highlight all sequences detected in this study and indicate the origin of each isolate. The scale bar at the bottom visualizes the number of substitutions per site. Evolutionary analyses were conducted by MEGA X [50].

Our calculation of a phylogenetic tree with BMMF reference sequences showed that most feces-derived BMMF molecules (14 out of 20) clustered in BMMF group 2, comprising Sphinx 2.36-like DNA elements (Figure 2). This further contrasts results from water buffalo, sheep and goat milk, where the majority of the isolates belonged to BMMF group 1 [35,36]. The remaining six BMMF isolates from the present study exhibited highest similarities to BMMF1 entities. Overall, the phylogenetic tree calculated for BMMF revealed a heterogeneous distribution (Figure 2). Sequences originating from animals kept at the same location were, in most cases, only distantly related. This broad distribution differs from the results obtained earlier from water buffalo milk, where isolates from the same flock formed clusters [35]. However, sequence alignments revealed that isolates WB3FI1 and WB4FI1 originating from two water buffaloes kept together in zoo C differed in a few nucleotides only. Remarkably, the fecal isolate Y1FI1 from a yak housed in zoo A hundreds of kilometers away from zoo C resembled these two isolates (WB3FI1 and WB4FI1). These three molecules cluster closely to elements isolated earlier from water buffalo and goat milk. Similarly, high sequence homologies could be observed comparing the isolates Z1FI1 with WB3FI3 and Z5FI1 with WB2FI3. The remaining fecal BMMF molecules shared sequence similarities ranging from 40% to >90% identity. Altogether, these data underline the BMMF diversity within the different locations and within a single animal. Although the number of animals under study was limited, the results indicate that fecal matter is an important source of BMMF DNA. The frequent detection of BMMF in feces evokes the question of whether these molecules are more likely to be associated with plants, environmental or gut bacteria than mammals.

The typical genome organization of BMMF was first described by zur Hausen et al. [6] and de Villiers et al. [5]. In silico analyses of our isolates showed the presence of undivided *rep*-genes except for isolate Z1FI2 (Figure 1). The latter features three separate ORFs starting an ‘ATG’ and encoding Reps highly similar to *Acinetobacter* replication proteins (Supplementary Table S1). In previous studies, de Villiers et al. fully characterized BMMF2 molecules in silico and discovered Rep proteins compiled of truncated ORFs [5]. Up to four additional ORFs were detected in all genomes (except for WB2FI1) upon the application of the ORFfinder software (Figure 1). To clarify whether these ORFs result in functional proteins or whether splicing events occur during RNA processing, further experiments are required.

An important characteristic of all BMMF1 elements is the presence of a TR region upstream of the *rep*-gen that is missing in all other BMMF groups. The TR consists of 22 nt that are repeated three times completely and once incompletely. Approx. 50 nt upstream of the TR region all BMMF1 molecules feature a highly conserved palindromic IR (TAAATGCTTTTA) that is probably the origin of replication (Table 3). Both TR and IR represent structural elements required for DNA recognition by the Rep [5,6].

#### 3.1.2. Isolation of CRESS DNA Viruses from Feces

**Figure 3:**
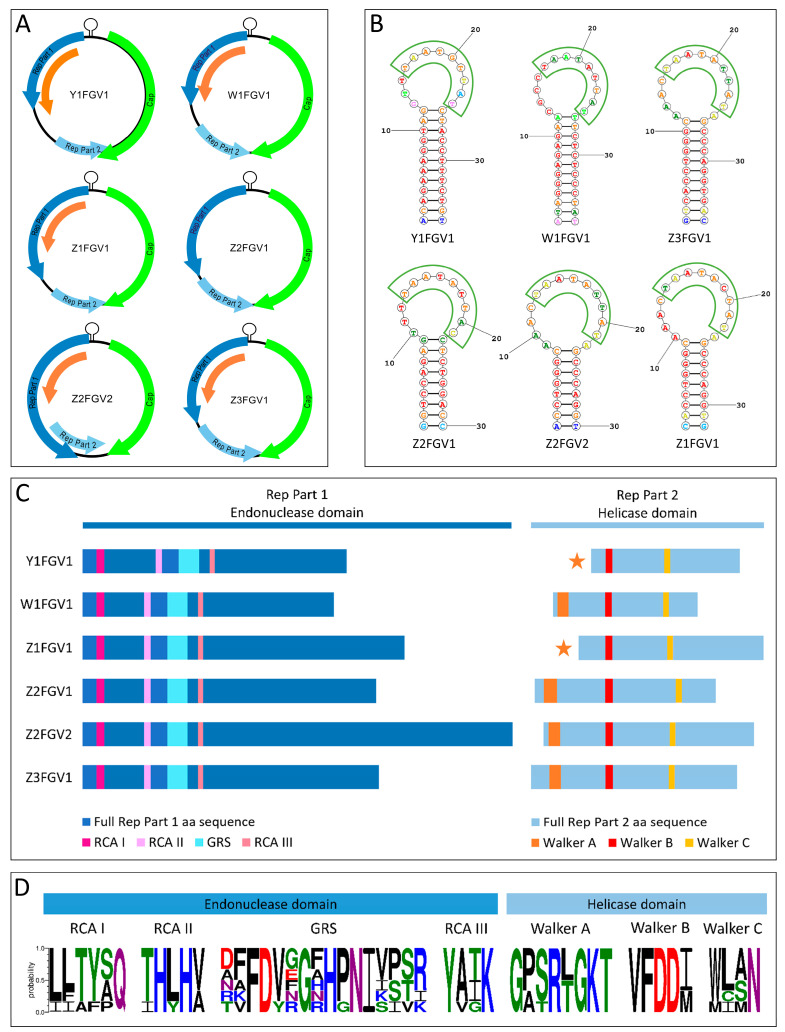
In-depth characterization of genomovirus sequences. (**A**) Genomic organization of six genomovirus sequences. Dark blue arrows represent the first part (Rep Part 1) of a potential *rep*-gene, while light blue arrows show the second part (Rep Part 2). *Cap*-genes are illustrated by green and further hypothetical ORFs by orange arrows. (**B**) Predicted stem-loop structure of each genomovirus, including the nonanucleotide motif that is highlighted by a green border (https://rna.urmc.rochester.edu/ accessed on 10 March 2023). (**C**) Illustration of the *rep*-gene organization with conserved endonuclease and helicase amino acid motifs. ORFs named ‘Rep Part 1’ contained the RCA motifs I, II, III and the GRS domain. Except for isolates Y1FGV1 and Z1FGV1, ‘Rep Part 2’ exhibited the motifs Walker A, B and C. In the two exceptions, the Walker A motif was located in the non-coding region between ‘Rep Part 1’ and ‘Rep Part 2’, indicated by the orange star. (**D**) Depiction of the variation within the amino acid motifs present in the six genomoviruses using WebLogo 3.7.12 (https://weblogo.threeplusone.com/create.cgi accessed on 10 March 2023). Colors indicate chemical properties of the different residues (black: hydrophobic; green: polar; purple: neutral; blue: basic; red: acidic).

Unlike BMMF entities, genomoviruses belong to the virus phylum *Cressdnaviricota* and are therefore officially classified as viruses by the International Committee on Taxonomy of Viruses (ICTV) [19]. The family *Genomoviridae* currently consists of ten genera, including 237 different species. Although CRESS viruses are detectable in a large number of diverse samples and animals [23,51,52], little is known about the occurrence and biology of Genomoviruses in farm animals [8,35,36,37,53]. This study confirms the observation of other authors reporting the frequent presence of genomoviruses in feces [26,54,55]. Six different genomovirus sequences were isolated from 5 out of 16 individuals. Three of these animals (Y1, W1, Z1) were also confirmed to be BMMF positive in feces (Table 2). The six genomovirus sequences (Figure 3) were characterized according to the recommendations of Varsani and Krupovic [9]. Typically, genomoviruses exhibit insert-bearing *rep*-genes [27,56]. It is also known that encoded Reps contain conserved amino acid motifs associated with their endonuclease and helicase function [9]. The variation of amino acids within these conserved motifs of the six molecules detected in this study is shown in Figure 3. The isolated genomes comprised two ORFs encoding Rep Part 1 and Rep Part 2 (Figure 3). Rep Part 1 exhibited the typical amino acid motifs RCA I, II, III and GRS, while Rep Part 2 contained the helicase motifs Walker A, B and C. Figure 3 further showed an uncommon, potential *rep*-translation product for isolates Y1FGV1 and Z1FGV1. This probably resulted in Walker A, located in the non-coding region between Rep Part 1 and Rep Part 2. However, whether this peculiarity resulted in impaired Rep expression could not be clarified in the present study. Furthermore, Rep Part 2 of the isolates W1FGV1, Z2FGV2 and Z3FGV1 included the alternative start codon ‘CTG’ and therefore differed from Rep Part 1, starting on ‘ATG’ in all six genomes. In complementary sense direction to *rep*, an ORF encoding a capsid protein (Cap) was detected in all isolates. This classic feature of *Genomoviridae* family members was first described by Varsani and Krupovic [19]. *Rep* and *cap* are separated by a stem-loop structure containing a conserved nonanucleotide motif at the loop tip (Figure 3).

**Figure 4:**
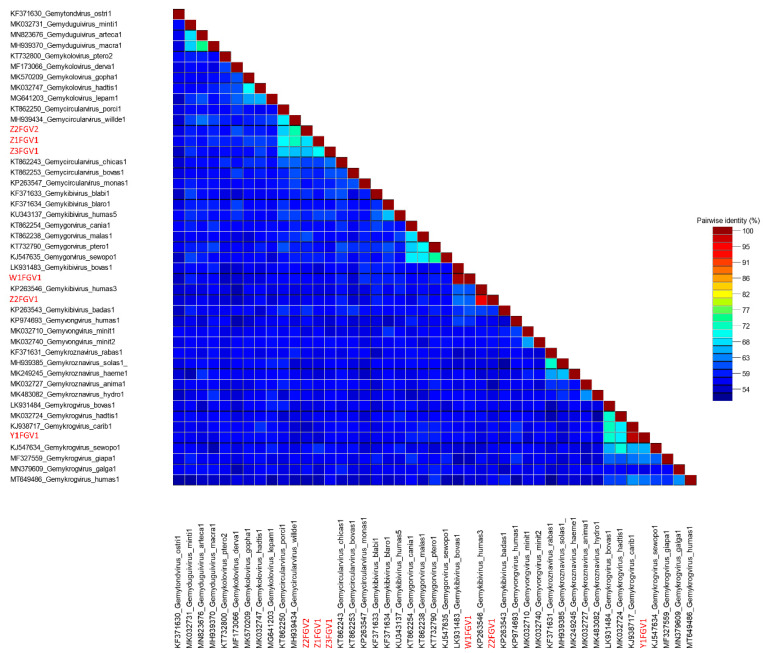
Genome-wide pairwise nucleotide similarity score matrix, including six full-length genomoviruses from this study and ICTV-listed representatives of the family *Genomoviridae* (except for the genus *Gemytripvirus*). Sequences from this study are written in red letters.

**Figure 5:**
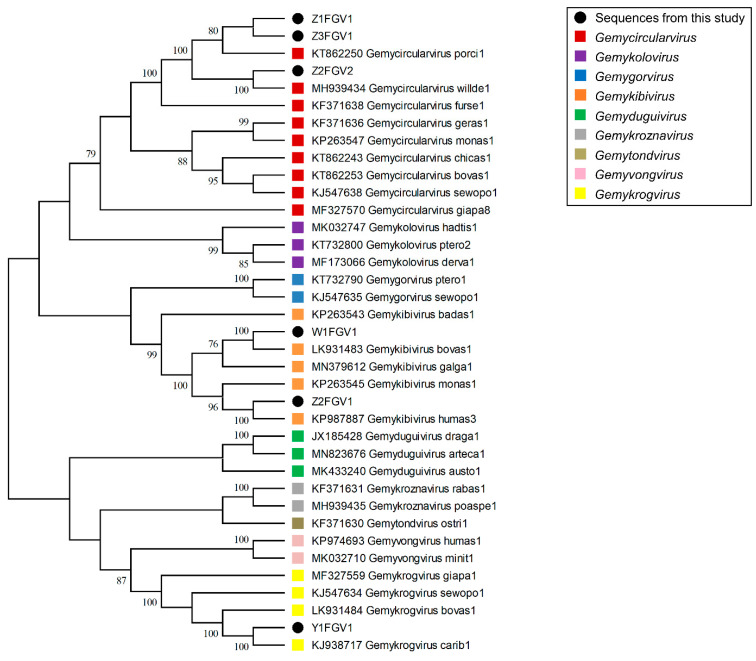
Maximum Likelihood phylogenetic tree of the six identified genomoviruses and representatives of the genomovirus genera (except for the genus *Gemytripvirus*). The evolutionary history was inferred by using the Maximum Likelihood method and the Tamura-Nei model [49]. The bootstrap consensus tree is based on 500 replicates. The percentage of trees in which the associated taxa clustered together is shown next to the branches. Branch support values lower than 70% were not included. Black dots highlight all sequences detected in this study. The colored squares indicate the different genomovirus genera. Evolutionary analyses were conducted by MEGA X [50].

Calculation of the pairwise genome-wide nucleotide similarity score and the resulting SDT matrix (Figure 4) of the novel molecules in comparison to ICTV-listed genomovirus reference genomes enabled their assignment to classified species [9]. Additionally, conserved amino acid and nonanucleotide motifs (Table 4) allowed us to categorize isolates W1FGV1 and Z2FGV1 as gemykibiviruses. Due to the species demarcation threshold of 78% [19], W1FGV1 represented a variant of the ICTV listed species *Gemykibivirus bovas1* (LK931483), with 99% pairwise identity [8]. The isolate Z2FGV1 showed highest similarities to *Gemykibivirus humas3* (KP263546), with 96.5% identity [57]. At 98%, the isolate Y1FGV1 was deemed to be a variant of the species *Gemykrogvirus carib1* (KJ938717) [58]. Interestingly, three genomoviruses detected in fecal samples (Z1FGV1, Z2FGV2 and Z3FGV1) shared 68–75% nucleotide pairwise identity with their closest related ICTV-listed genomovirus, listed as *Gemycircularvirus willde1* (MH939434) (Figure 4). Isolate Z3FGV1 shared 74% pairwise identity to an unclassified genomovirus sequence from a wild bird which is available in GenBank (MW183023) but not included in the SDT matrix. Among themselves, these three isolates share 67–71% pairwise identity. Calculation of a phylogenetic tree including genomovirus reference genomes pointed to a relationship to gemycircularviruses (Figure 5). Additionally, the phylogenetic analysis of Reps supported the assignment to the genus *Gemycircularvirus* (Supplementary Figure S1). Based on the proposed demarcation threshold [9], the three isolates (Z1FGV1, Z2FGV2 and Z3FGV1) might be considered to represent novel species in this genus.

### 3.2. Blood and Serum Samples

The analysis of fecal samples widens the general knowledge on the occurrence of circular DNA but provides no information about a potential uptake of these molecules into the bloodstream. Therefore, in addition to fecal samples, 24 fresh EDTA-blood samples and 36 frozen serum samples from animals kept in German zoos were analyzed for BMMF molecules.

In contrast to other studies reporting on the successful isolation of BMMF from taurine cattle serum [11,29], all serum samples in the present study tested negative for BMMF. Three out of 24 EDTA-blood samples contained full-length BMMF sequences, with two of them originating from yaks (Y1BI1, Y2BI1) and one from a water buffalo (WB1BI1). In contrast to the heterogeneous feces-derived BMMF molecules, these three blood isolates were all BMMF1. They shared 77–83% nucleotide identity among themselves and formed a common clade in the phylogenetic tree (Figure 2). Currently, modes of BMMF uptake and transmission are still unclear. Beside ingestion with forage, it is also conceivable that BMMF might be transmitted via vectors like insects or ticks. As ‘exotic’ bovine species in zoos are often housed together with other animals, an inter-species transmission of BMMF is imaginable. Y2BI1 and WB1BI1 are highly similar to BMMF molecules isolated from water buffalo milk. The Y1BI1 isolate had high nucleotide homologies to BMMF1 molecule CMI2.214 [11] and to an *Acinetobacter* sp. plasmid (Supplementary Table S1). Furthermore, it featured some molecular characteristics that differed from most of the BMMF1 molecules in this study. An in silico search for ORFs revealed two separate, relatively short ORFs, named RepA and RepB, encoding a replicase. This feature resembled the genome organization of many BMMF2 molecules [5]. The putative Rep proteins of Y1BI1 started on ‘TTG’ (RepA) and on ‘CTG’ (RepB). In contrast to other isolates in which the TR is located approx. 60 nts upstream of the *rep*-gene, the TR of Y1BI1 could be found directly upstream of RepA. Another difference was the composition of the TR. In this particular case, it started with a partial repeat of 14 nts and proceeded with three direct repeats consisting of 22 nts (Table 3). As these data were obtained from in silico analyses only, it cannot be concluded whether this BMMF sequence is replication competent or not.

### 3.3. Matched Blood and Fecal Samples

An initial objective of the current project was to test Asian and African cattle for the presence of BMMF or CRESS DNA viruses. As the occurrence of these entities in feces only does not provide information on potential systemic circulation, it is necessary to comparatively examine feces and blood from individual animals. Matched samples were available from 15 of the 16 aforementioned animals.

#### 3.3.1. BMMF in Blood and Feces

Only one individual yak (Y1) tested positive for BMMF genomes in both feces and blood. Interestingly, in addition to one BMMF1 genome in blood (Table 2), the feces of the respective yak was found to contain a variety of diverse circular molecules (three BMMF and one genomovirus). The BMMF1 blood isolate Y1BI1 shared 81% nucleotide identity with the feces isolate Y1FI1. The remaining fecal BMMF genomes from this animal showed few similarities to Y1BI1. One further striking finding regarding matched blood and fecal samples applies to animal WB1. The blood sample of this individual contained a BMMF1 molecule (WB1BI1) that has been described above (3.2 Blood and Serum Samples). Although the fecal sample of this animal tested positive for short BMMF fragments during PCR screening, amplification of circular, full-length genomes failed. This phenomenon has already been observed during analyses of water buffalo, sheep and goat milk [35,36]. At the current state of knowledge, we cannot assess whether the circulation of BMMF entities in the bloodstream can cause disease in animals or not. Furthermore, we were unable to determine the pathways on which BMMF enter the bloodstream and where replication occurs. To clarify this, replication surveys in different cell lines and Rep antigen detection in animal tissues would be highly desirable.

#### 3.3.2. CRESS DNA Viruses in Blood and Feces

The absence of genomoviruses in the blood of apparently healthy animals, despite testing positive in feces, indicates that the resorption of genomovirus DNA seems to be a rare event [37,59]. The exclusive occurrence of genomovirus isolates in feces supports the hypothesis that these entities might have been ingested with forage plants, thus reaching the gastrointestinal tract. Several earlier publications have demonstrated the common occurrence of genomoviruses in animal feces and discussed the role of animals’ diets [20,21,22,57,60]. Since there are no confirmed hosts for most members of the family *Genomoviridae*, it is conceivable that these viruses infect fungi and/or plants that serve as animal food [61].

A further interpretation might be that bovine species do not belong to the typical host range of these viruses but sometimes may serve as transient hosts. The transmission of CRESS DNA viruses via vectors has already been hypothesized [62,63,64]. However, the discrepancy between blood and feces results could have arisen from collecting blood samples during a “non-viremic” phase [37]. To clarify this, further investigations need to be initiated with consecutive regular blood sampling over time.

Other studies focusing on circular ssDNA viruses (e.g., *Anelloviridae* and *Circoviridae*) in farm animals have reported a much higher prevalence compared to our results [65,66]. This discrepancy might be attributed to some limitations in this study, such as the sample size and the use of targeted primers. Therefore, the frequency and diversity of circular DNA viruses and BMMF could potentially be higher than shown here. The application of alternative and supportive methods like Next Generation Sequencing could provide clarification in this respect.

## 4. Conclusions

Although BMMF have frequently been isolated from cow, sheep and goat milk at retail, as well as from beef, their presence in other *Bovidae* family members that also play an important role in human nutrition worldwide was previously unknown. The detection of BMMF DNA and Rep protein in colon tissues of human cancer patients stresses the importance of studies on BMMF occurrence in food-producing animals. Herein, we isolated 23 BMMF molecules (14 × BMMF2, 9 × BMMF1) from yak, zebu, watusi cattle and water buffalo. Most BMMF sequences originated from feces (14 × BMMF2, 6 × BMMF1), whereas only three BMMF1 elements were isolated from blood. Additionally, six sequences belonging to the virus family *Genomoviridae* were isolated from fecal matter. Due to intrinsic limitations, questions on the specific distribution of BMMF in *Bovidae*, including the differentiation of wild-life and zoo animals and elucidation of the route of transmission (forage, bacteria etc.), remain to be addressed in future studies.

## Figures and Tables

**Table 1:** Overview of samples analyzed in this study. All animals were kept in nine different German zoos.

Species/Breed	Serum	EDTA-Blood	Feces	No. of Individuals
Watusi	22	6	4 *	28
Zebu	13	10	6 *	23
Yak	1	4	2 *	5
Water buffalo	-	4	3 * + 1	5

* indicates that beside feces, EDTA-blood or serum from the corresponding animal was available.

**Table 2:** List of all 29 circular ssDNA isolates identified in EDTA-blood and fecal samples of different bovids from three zoos. No circular entities were found in animals from the remaining six zoos. Nt = nucleotides.

Individual	Species/Breed	Zoo/Origin	Isolated Molecules	Length in Nt	Acc. No.	Sample Material	DNA-Type
Y1	Yak	A	Y1FI1	2522	OQ633422	Feces	BMMF1
			Y1FI2	2387	OQ633423	Feces	BMMF2
			Y1FI3	2346	OQ633424	Feces	BMMF2
			Y1FGV1	2232	OQ633425	Feces	*Genomoviridae*
			Y1BI1	1616	OQ633426	EDTA-blood	BMMF1
Y2	Yak	A	Y2BI1	1959	OQ633427	EDTA-blood	BMMF1
Y3	Yak	A	Y3FI1	2229	OQ633428	Feces	BMMF2
W1	Watusi	B	W1FI1	2238	OQ633429	Feces	BMMF2
			W1FGV1	2134	OQ633430	Feces	*Genomoviridae*
Z1	Zebu	B	Z1FI1	2297	OQ633431	Feces	BMMF2
			Z1FI2	2733	OQ633432	Feces	BMMF2
			Z1FI3	2146	OQ633433	Feces	BMMF1
			Z1FGV1	2215	OQ633434	Feces	*Genomoviridae*
Z2	Zebu	B	Z2FGV1	2118	OQ633435	Feces	*Genomoviridae*
			Z2FGV2	2216	OQ633436	Feces	*Genomoviridae*
Z3	Zebu	B	Z3FGV1	2200	OQ633437	Feces	*Genomoviridae*
Z4	Zebu	B	Z4FI1	2353	OQ633438	Feces	BMMF2
			Z4FI2	2118	OQ633439	Feces	BMMF1
Z5	Zebu	B	Z5FI1	2280	OQ633440	Feces	BMMF2
WB1	Water Buffalo	C	WB1BI1	2185	OQ633441	EDTA-blood	BMMF1
WB2	Water Buffalo	C	WB2FI1	1913	OQ633442	Feces	BMMF1
			WB2FI2	2476	OQ633443	Feces	BMMF2
			WB2FI3	2375	OQ633444	Feces	BMMF2
			WB2FI4	2447	OQ633445	Feces	BMMF2
WB3	Water Buffalo	C	WB3FI1	2521	OQ633446	Feces	BMMF1
			WB3FI2	2359	OQ633447	Feces	BMMF2
			WB3FI3	2298	OQ633448	Feces	BMMF2
WB4	Water Buffalo	C	WB4FI1	2522	OQ633449	Feces	BMMF1
			WB4FI2	2380	OQ633450	Feces	BMMF2

Abbreviations: Y = Yak, W = Watusi, Z = Zebu, WB = Water Buffalo, BI = Blood Isolate, FI = Feces Isolate, FGV = Feces Genomovirus.

**Table 3:** Overview of the six BMMF1 molecules with respective tandem repeats (TR) and inverted repeats (IR). The horizontal line separates fecal from blood isolates. The last column indicates the number of nucleotides (Nt) between TR and IR.

Isolate	Period Size	TR	IR	Nt between TR and IR
Y1FI1	22	ATACCCCTACGTTTACCGATCA	TAAATGCTTTTA	50
Z1FI3	22	ATACTCCTAGGTTTACCTACCA	TAAATGCTTTTA	50
Z4FI2	22	CTACGTTTACCCATCAATACCC	TAAATGCTTTTA	56
WB2FI1	22	CCTACGTTTACCGATCAATACC	TAAATGCTTTTA	55
WB3FI1	22	ATACCCCTACGTTTACCGATCA	TAAATGCTTTTA	50
WB4FI1	22	ATACCCCTACGTTTACCGATCA	TAAATGCTTTTA	50
Y1BI1	22	TACCAATACTCCTAGGTTTACC	TAAATGCTTTTA	53
Y2BI1	22	CACCGTTTACCCATCAATATGA	TAAATGCTTTTA	56
WB1BI1	22	ATACTCCTAGGTTTACCTACCA	TAAATGCTTTTA	50

**Table 4:** Overview of conserved nonanucleotide motifs and amino acid motifs of all genomoviruses identified in this study. Background colors indicate their relation to different genomovirus genera (yellow: *Gemykrogvirus*; blue: *Gemycircularvirus*; green: *Gemykibivirus*). Red letters indicate two motifs located in the intergenic region between Rep Part 1 and Rep Part 2.

Isolate	Nonanucleotide	Motif I	Motif II	GRS Motif	Motif III	Walker A	Walker B	Walker C
Y1FGV1	TAATATTAT	IITFPQ	IHYHV	TAFDYFGAHGNIKSVR	YVGK	GPTRTGKT	VFDDI	MCMN
Z1FGV1	TAATACTAT	LLAYAQ	THLHV	DFFDVGGHHPNIVPSR	YATK	GASRLGKT	VFDDM	WLAN
Z2FGV2	TAATATTAT	LLTYSQ	THLHV	DFFDVNGNHPNIVPSR	YATK	GPSRLGKT	VFDDI	WLAN
Z3FGV1	TAATATTAT	LLTYSQ	THLHV	NFFDVRGRHPNIVPSR	YAIK	GASRLGKT	VFDDI	WLAN
W1FGV1	TAATGTTAT	LLTYAQ	THLHA	AVFDVGGFHPNISITK	YAIK	GPSRTGKT	VFDDI	WISN
Z2FGV1	TAATATTAC	LFTYSQ	THLHA	RKFDVEGFHPNIISTI	YATK	GPSRTGKT	VFDDM	WLSN

## Data Availability

The data presented in this study are available within the article and the supplementary material. There are 29 nucleotide sequences accessible at NCBI GenBank database (Accession numbers OQ633422-OQ633450).

## Supplementary Materials

The following are available online at https://www.mdpi.com/article/10.3390/ani13091492/s1, Figure S1: Maximum Likelihood phylogenetic tree of the identified Rep amino acid sequences from this study and representatives of nine genomoviral genera. Table S1: Summary of Blast results.

## References

[1] Faria F., Filho A., Madalena F., Josahkian L. (2009). Pedigree analysis in the Brazilian Zebu breeds. J. Anim. Breed. Genet..

[2] Leslie D.M., Schaller G.B. (2009). *Bos grunniens* and *Bos mutus* (Artiodactyla: Bovidae). Mamm. Species.

[3] Kugonza D., Nabasirye M., Mpairwe D., Hanotte O., Okeyo A. (2011). Productivity and morphology of Ankole cattle in three livestock production systems in Uganda. Anim. Genet. Resour./Resour. Génétiques Anim./Recur. Genéticos Anim..

[4] Pasha T., Hayat Z. (2012). Present situation and future perspective of buffalo production in Asia. J. Anim. Plant Sci..

[5] de Villiers E.M., Gunst K., Chakraborty D., Ernst C., Bund T., zur Hausen H. (2019). A specific class of infectious agents isolated from bovine serum and dairy products and peritumoral colon cancer tissue. Emerg. Microbes Infect..

[6] zur Hausen H., Bund T., de Villiers E.M., Hunter E., Bister K., Compans R.W. (2017). Infectious Agents in Bovine Red Meat and Milk and Their Potential Role in Cancer and Other Chronic Diseases. Viruses, Genes, and Cancer.

[7] Manuelidis L. (2011). Nuclease resistant circular DNAs copurify with infectivity in scrapie and CJD. J. Neurovirol..

[8] Lamberto I., Gunst K., Muller H., zur Hausen H., de Villiers E.M. (2014). Mycovirus-like DNA virus sequences from cattle serum and human brain and serum samples from multiple sclerosis patients. Genome Announc..

[9] Varsani A., Krupovic M. (2017). Sequence-based taxonomic framework for the classification of uncultured single-stranded DNA viruses of the family Genomoviridae. Virus Evol..

[10] Gunst K., zur Hausen H., de Villiers E.M. (2014). Isolation of bacterial plasmid-related replication-associated circular DNA from a serum sample of a multiple sclerosis patient. Genome Announc..

[11] Whitley C., Gunst K., Muller H., Funk M., zur Hausen H., de Villiers E.M. (2014). Novel replication-competent circular DNA molecules from healthy cattle serum and milk and multiple sclerosis-affected human brain tissue. Genome Announc..

[12] Falida K., Eilebrecht S., Gunst K., zur Hausen H., de Villiers E.M. (2017). Isolation of Two Virus-Like Circular DNAs from Commercially Available Milk Samples. Genome Announc..

[13] Gilbert W., Dressler D. (1968). DNA replication: The rolling circle model. Cold Spring Harb. Symp. Quant. Biol..

[14] Dressler D., Wolfson J. (1970). The Rolling Circle for ϕX DNA Replication, III. Synthesis of Supercoiled Duplex Rings. Proc. Natl. Acad. Sci..

[15] Baas P.D. (1985). DNA replication of single-stranded Escherichia coli DNA phages. Biochim. Et Biophys. Acta (BBA)—Gene Struct. Expr..

[16] Koonin E.V., Ilyina T.V. (1992). Geminivirus replication proteins are related to prokaryotic plasmid rolling circle DNA replication initiator proteins. J. Gen. Virol..

[17] Ruiz-Masó J.A., MachóN C., Bordanaba-Ruiseco L., Espinosa M., Coll M., Del Solar G. (2015). Plasmid Rolling-Circle Replication. Microbiol. Spectr..

[18] Krupovic M., Varsani A., Kazlauskas D., Breitbart M., Delwart E., Rosario K., Yutin N., Wolf Y.I., Harrach B., Zerbini F.M. (2020). Cressdnaviricota: A Virus Phylum Unifying Seven Families of Rep-Encoding Viruses with Single-Stranded, Circular DNA Genomes. J. Virol..

[19] Varsani A., Krupovic M. (2021). Family Genomoviridae: 2021 taxonomy update. Arch. Virol..

[20] Sikorski A., Massaro M., Kraberger S., Young L.M., Smalley D., Martin D.P., Varsani A. (2013). Novel myco-like DNA viruses discovered in the faecal matter of various animals. Virus Res..

[21] Levy H., Fontenele R.S., Harding C., Suazo C., Kraberger S., Schmidlin K., Djurhuus A., Black C.E., Hart T., Smith A.L. (2020). Identification and Distribution of Novel Cressdnaviruses and Circular Molecules in Four Penguin Species in South Georgia and the Antarctic Peninsula. Viruses.

[22] Orton J.P., Morales M., Fontenele R.S., Schmidlin K., Kraberger S., Leavitt D.J., Webster T.H., Wilson M.A., Kusumi K., Dolby G.A. (2020). Virus discovery in desert tortoise fecal samples: Novel circular single-stranded DNA viruses. Viruses.

[23] Tisza M.J., Pastrana D.V., Welch N.L., Stewart B., Peretti A., Starrett G.J., Pang Y.S., Krishnamurthy S.R., Pesavento P.A., McDermott D.H. (2020). Discovery of several thousand highly diverse circular DNA viruses. eLife.

[24] Cibulski S., Alves de Lima D., Fernandes Dos Santos H., Teixeira T.F., Tochetto C., Mayer F.Q., Roehe P.M. (2021). A plate of viruses: Viral metagenomics of supermarket chicken, pork and beef from Brazil. Virology.

[25] Wiederkehr M.A., Qi W., Schoenbaechler K., Fraefel C., Kubacki J. (2022). Virus Diversity, Abundance, and Evolution in Three Different Bat Colonies in Switzerland. Viruses.

[26] Custer J.M., White R., Taylor H., Schmidlin K., Fontenele R.S., Stainton D., Kraberger S., Briskie J.V., Varsani A. (2022). Diverse single-stranded DNA viruses identified in New Zealand (Aotearoa) South Island robin (*Petroica australis*) fecal samples. Virology.

[27] Krupovic M., Ghabrial S.A., Jiang D., Varsani A. (2016). Genomoviridae: A new family of widespread single-stranded DNA viruses. Arch. Virol..

[28] Kazlauskas D., Varsani A., Koonin E.V., Krupovic M. (2019). Multiple origins of prokaryotic and eukaryotic single-stranded DNA viruses from bacterial and archaeal plasmids. Nat. Commun..

[29] Funk M., Gunst K., Lucansky V., Muller H., zur Hausen H., de Villiers E.M. (2014). Isolation of protein-associated circular DNA from healthy cattle serum. Genome Announc..

[30] zur Hausen H., Bund T., de Villiers E.M. (2018). Specific nutritional infections early in life as risk factors for human colon and breast cancers several decades later. Int. J. Cancer.

[31] Bund T., Nikitina E., Chakraborty D., Ernst C., Gunst K., Boneva B., Tessmer C., Volk N., Brobeil A., Weber A. (2021). Analysis of chronic inflammatory lesions of the colon for BMMF Rep antigen expression and CD68 macrophage interactions. Proc. Natl. Acad. Sci. USA.

[32] de Villiers E.M., zur Hausen H. (2021). Bovine Meat and Milk Factors (BMMFs): Their Proposed Role in Common Human Cancers and Type 2 Diabetes Mellitus. Cancers.

[33] Nikitina E., Alikhanyan K., Neßling M., Richter K., Kaden S., Ernst C., Seitz S., Chuprikova L., Häfele L., Gunst K. (2022). Structural Expression of BMMF in tissues of colorectal, lung and pancreatic cancer patients. Int. J. Cancer.

[34] Nikitina E., Burk-Körner A., Wiesenfarth M., Alwers E., Heide D., Tessmer C., Ernst C., Krunic D., Schrotz-King P., Chang-Claude J. (2023). Bovine meat and milk factor protein expression in tumor-free mucosa of colorectal cancer patients coincides with macrophages and might interfere with patient survival. Mol. Oncol..

[35] König M.-T., Fux R., Link E., Sutter G., Märtlbauer E., Didier A. (2021). Circular Rep-Encoding Single-Stranded DNA Sequences in Milk from Water Buffaloes (*Bubalus arnee* f. *bubalis*). Viruses.

[36] König M.-T., Fux R., Link E., Sutter G., Märtlbauer E., Didier A. (2021). Identification and Characterization of Circular Single-Stranded DNA Genomes in Sheep and Goat Milk. Viruses.

[37] Lechmann J., Ackermann M., Kaiser V., Bachofen C. (2021). Viral infections shared between water buffaloes and small ruminants in Switzerland. J. Vet. Diagn. Investig..

[38] Pohl S., Habermann D., Link E.K., Fux R., Boldt C.L., Franz C.M.A.P., Hölzel C., Klempt M. (2022). Detection of DNA sequences attributed to bovine meat and milk factors (BMMF/SPHINX) in food-related samples. Food Control.

[39] Scherf B., Pilling D. (2015). The Second Report on the State of the World’s Animal Genetic Resources for Food and Agriculture.

[40] Zhang K., Lenstra J.A., Zhang S., Liu W., Liu J. (2020). Evolution and domestication of the Bovini species. Anim. Genet..

[41] Johnson M., Zaretskaya I., Raytselis Y., Merezhuk Y., McGinnis S., Madden T.L. (2008). NCBI BLAST: A better web interface. Nucleic Acids Res..

[42] Tamura K., Peterson D., Peterson N., Stecher G., Nei M., Kumar S. (2011). MEGA5: Molecular evolutionary genetics analysis using maximum likelihood, evolutionary distance, and maximum parsimony methods. Mol. Biol. Evol..

[43] Burland T.G., Misener S., Krawetz S.A. (2000). DNASTAR’s Lasergene Sequence Analysis Software. Bioinformatics Methods and Protocols.

[44] Edgar R.C. (2004). MUSCLE: Multiple sequence alignment with high accuracy and high throughput. Nucleic Acids Res..

[45] Muhire B.M., Varsani A., Martin D.P. (2014). SDT: A Virus Classification Tool Based on Pairwise Sequence Alignment and Identity Calculation. PLoS ONE.

[46] The European Molecular Biology Open Software Suite (EMBOSS) A. http://emboss.bioinformatics.nl/cgi-bin/emboss/equicktandem.

[47] The European Molecular Biology Open Software Suite (EMBOSS) B. https://www.bioinformatics.nl/cgi-bin/emboss/palindrome.

[48] National Center for Biotechnology Information (NCBI) ORFfinder. https://www.ncbi.nlm.nih.gov/orffinder/.

[49] Tamura K., Nei M. (1993). Estimation of the number of nucleotide substitutions in the control region of mitochondrial DNA in humans and chimpanzees. Mol. Biol. Evol..

[50] Kumar S., Stecher G., Li M., Knyaz C., Tamura K. (2018). MEGA X: Molecular evolutionary genetics analysis across computing platforms. Mol. Biol. Evol..

[51] Rosario K., Mettel K.A., Benner B.E., Johnson R., Scott C., Yusseff-Vanegas S.Z., Baker C.C.M., Cassill D.L., Storer C., Varsani A. (2018). Virus discovery in all three major lineages of terrestrial arthropods highlights the diversity of single-stranded DNA viruses associated with invertebrates. PeerJ.

[52] Zhao L., Rosario K., Breitbart M., Duffy S. (2019). Eukaryotic Circular Rep-Encoding Single-Stranded DNA (CRESS DNA) Viruses: Ubiquitous Viruses With Small Genomes and a Diverse Host Range. Adv. Virus Res..

[53] Ling Y., Zhang X., Qi G., Yang S., Jingjiao L., Shen Q., Wang X., Cui L., Hua X., Deng X. (2019). Viral metagenomics reveals significant viruses in the genital tract of apparently healthy dairy cows. Arch. Virol..

[54] Kraberger S., Waits K., Ivan J., Newkirk E., VandeWoude S., Varsani A. (2018). Identification of circular single-stranded DNA viruses in faecal samples of Canada lynx (*Lynx canadensis*), moose (*Alces alces*) and snowshoe hare (*Lepus americanus*) inhabiting the Colorado San Juan Mountains. Infect. Genet. Evol..

[55] Khalifeh A., Blumstein D.T., Fontenele R.S., Schmidlin K., Richet C., Kraberger S., Varsani A. (2021). Diverse cressdnaviruses and an anellovirus identified in the fecal samples of yellow-bellied marmots. Virology.

[56] Kraberger S., Argüello-Astorga G.R., Greenfield L.G., Galilee C., Law D., Martin D.P., Varsani A. (2015). Characterisation of a diverse range of circular replication-associated protein encoding DNA viruses recovered from a sewage treatment oxidation pond. Infect. Genet. Evol..

[57] Conceição-Neto N., Zeller M., Heylen E., Lefrère H., Mesquita J.R., Matthijnssens J. (2015). Fecal virome analysis of three carnivores reveals a novel nodavirus and multiple gemycircularviruses. Virol. J..

[58] Ng T.F.F., Chen L.-F., Zhou Y., Shapiro B., Stiller M., Heintzman P.D., Varsani A., Kondov N.O., Wong W., Deng X. (2014). Preservation of viral genomes in 700-y-old caribou feces from a subarctic ice patch. Proc. Natl. Acad. Sci. USA.

[59] Zhang W., Yang S., Shan T., Hou R., Liu Z., Li W., Guo L., Wang Y., Chen P., Wang X. (2017). Virome comparisons in wild-diseased and healthy captive giant pandas. Microbiome.

[60] Fontenele R.S., Lacorte C., Lamas N.S., Schmidlin K., Varsani A., Ribeiro S.G. (2019). Single stranded DNA viruses associated with capybara faeces sampled in Brazil. Viruses.

[61] Fontenele R.S., Roumagnac P., Richet C., Kraberger S., Stainton D., Aleamotu‘a M., Filloux D., Bernardo P., Harkins G.W., McCarthy J. (2020). Diverse genomoviruses representing twenty-nine species identified associated with plants. Arch. Virol..

[62] Ma R., Zhao M., Wang H., Hou R., Qin K., Qian Y., Zhang H., Zhou Y., Wu W., Gu J. (2022). Virome of Giant Panda-Infesting Ticks Reveals Novel Bunyaviruses and Other Viruses That Are Genetically Close to Those from Giant Pandas. Microbiol. Spectr..

[63] Waits K., Edwards M.J., Cobb I.N., Fontenele R.S., Varsani A. (2018). Identification of an anellovirus and genomoviruses in ixodid ticks. Virus Genes.

[64] Rosario K., Dayaram A., Marinov M., Ware J., Kraberger S., Stainton D., Breitbart M., Varsani A. (2012). Diverse circular ssDNA viruses discovered in dragonflies (Odonata: Epiprocta). J. Gen. Virol..

[65] Singh G., Ramamoorthy S. (2018). Potential for the cross-species transmission of swine torque teno viruses. Vet. Microbiol..

[66] Wang Y., Noll L., Lu N., Porter E., Stoy C., Zheng W., Liu X., Peddireddi L., Niederwerder M., Bai J. (2020). Genetic diversity and prevalence of porcine circovirus type 3 (PCV3) and type 2 (PCV2) in the Midwest of the USA during 2016–2018. Transbound. Emerg. Dis..

