# Supplementary material for: First Insights into the Occurrence of Circular Single-Stranded DNA Genomes in Asian and African Cattle
Source: Animals (Basel). 2023 Apr 27;13(9):1492. doi: 10.3390/ani13091492 (PMC10177065; doi:10.3390/ani13091492)
Supplement: Supplementary file 1 [file animals-13-01492-s001.zip › Supplementary Figure S1.pdf]

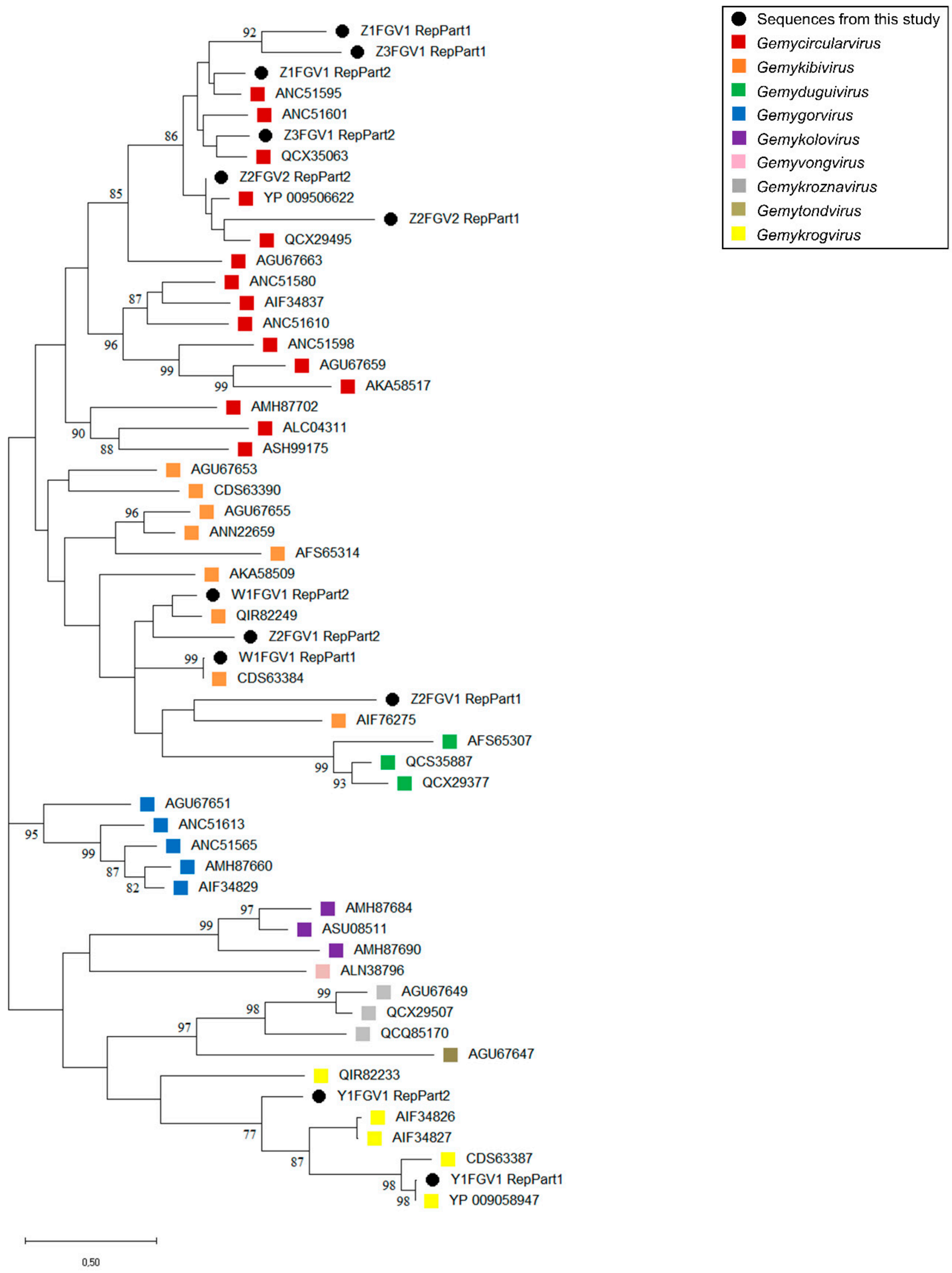

Supplementary Figure S1. Maximum Likelihood phylogenetic tree of the identified Rep amino acid sequences from this study and representatives of nine genomoviral genera. The evolutionary history was inferred by using the Maximum Likelihood method and Tamura-Nei model [49]. The bootstrap consensus tree is based on 500 replicates. The percentage of trees in which the associated taxa clustered together is shown next to the branches. Branch support values lower than 70% were not included. Black dots highlight all Reps detected in this study, the colored squares indicate the different genomoviral genera. The scale bar at the bottom visualizes the number of substitutions per site. Evolutionary analyses were computed by MEGA software.
